# Global Goat! Is the Expanding Goat Population an Important Reservoir of *Cryptosporidium*?

**DOI:** 10.3389/fvets.2021.648500

**Published:** 2021-03-05

**Authors:** Kjersti Selstad Utaaker, Suman Chaudhary, Tsegabirhan Kifleyohannes, Lucy Jane Robertson

**Affiliations:** ^1^Faculty of Bioscience and Aquaculture, Nord University, Bodø, Norway; ^2^Department of Pathology, School of Medicine, Case Western Reserve University, Cleveland, OH, United States; ^3^Parasitology, Department of Paraclinical Sciences, Faculty of Veterinary Medicine, Norwegian University of Life Sciences, Oslo, Norway; ^4^Department of Veterinary Basic and Diagnostic Sciences, College of Veterinary Medicine, Mekelle University, Mekelle, Ethiopia

**Keywords:** *Cryptosporidium*, goats (Capra aegagrus hircus), genotypes, One Health, zoonosis

## Abstract

Goats are a primary or additional income source for many families in resource-poor areas. Although often considered inferior to other livestock, the resilience of goats and their ability to thrive in a range of environments means that that they are of particular value. Furthermore, goats emit less methane than other livestock species. In these same areas, it is well-documented that cryptosporidiosis has a substantial impact on infant morbidity and mortality, as well as reducing child growth and development. As *Cryptosporidium* also causes diarrheal disease in goats, the question arises whether goats may represent a reservoir of infection to humans. Epidemiological studies regarding the potential for transmission of *Cryptosporidium* between goats and humans have largely concluded that *Cryptosporidium* species infecting goats are not zoonotic. However, these studies are mostly from developed countries, where goat husbandry is smaller, management routines differ greatly from those of developing countries, contact between goats and their owners is more limited, and cryptosporidiosis has less impact on human health. In this article, background information on goat husbandry in different countries is provided, along with information on *Cryptosporidium* prevalence among goats, at both the species and sub-species levels, and the potential for zoonotic transmission. The intention is to indicate data gaps that should be filled and to increase awareness of the role of goats as providers for low-income families, often living in areas where cryptosporidiosis is endemic and where appropriate baseline interventions could have a positive impact, regardless of species of goat or parasite.

## Introduction

Goats are one of the species of livestock that were domesticated earliest, and are used worldwide for milk, meat, and hair/skin. Nowadays, goats are among the most popular and beneficial livestock for those with limited resources (1). Small-scale goat production is of considerable benefit to families and communities globally, in a variety of climates and conditions.

A landmark paper from 2005, “Goats – pathway out of poverty,” argued that goats are worthy of serious investment, with the potential for transforming the lives of some of the world's poorest people (2). Even under extreme climate conditions, goats have several characteristics that enable their capacity to convert feed into milk and meat (3).

In a world where our future is increasingly dominated by adaptation to climate change, goat-keeping is emerging as a truly important husbandry, not only for maintaining production levels, but also due to its relatively minor impact on climate as goats emit less methane than other livestock (4). There are about one billion goats worldwide, and the global goat population has more than doubled during the last four decades. According to the Food and Agriculture Organization, over 90% of goats are found in developing countries; Asia has the largest proportion of the world's goat population, followed by Africa (5).

Goats are traditionally managed differently to cattle, with flocks grazing in expansive enclosures or not enclosed at all, rather than being kept indoors. Goats are also popular as backyard livestock for hard-pressed families with few resources since livestock accounts for up to 60% of their income (1). In these settings, barriers against animal-human-animal transmission of zoonotic diseases are weakened. Thus, in promoting and supporting goat farming, it is important that efforts are also made to ensure that transfer of pathogens between goats and their owners is minimized.

## Where Are the Goats, and Who Keeps Them?

**Figure 1:**
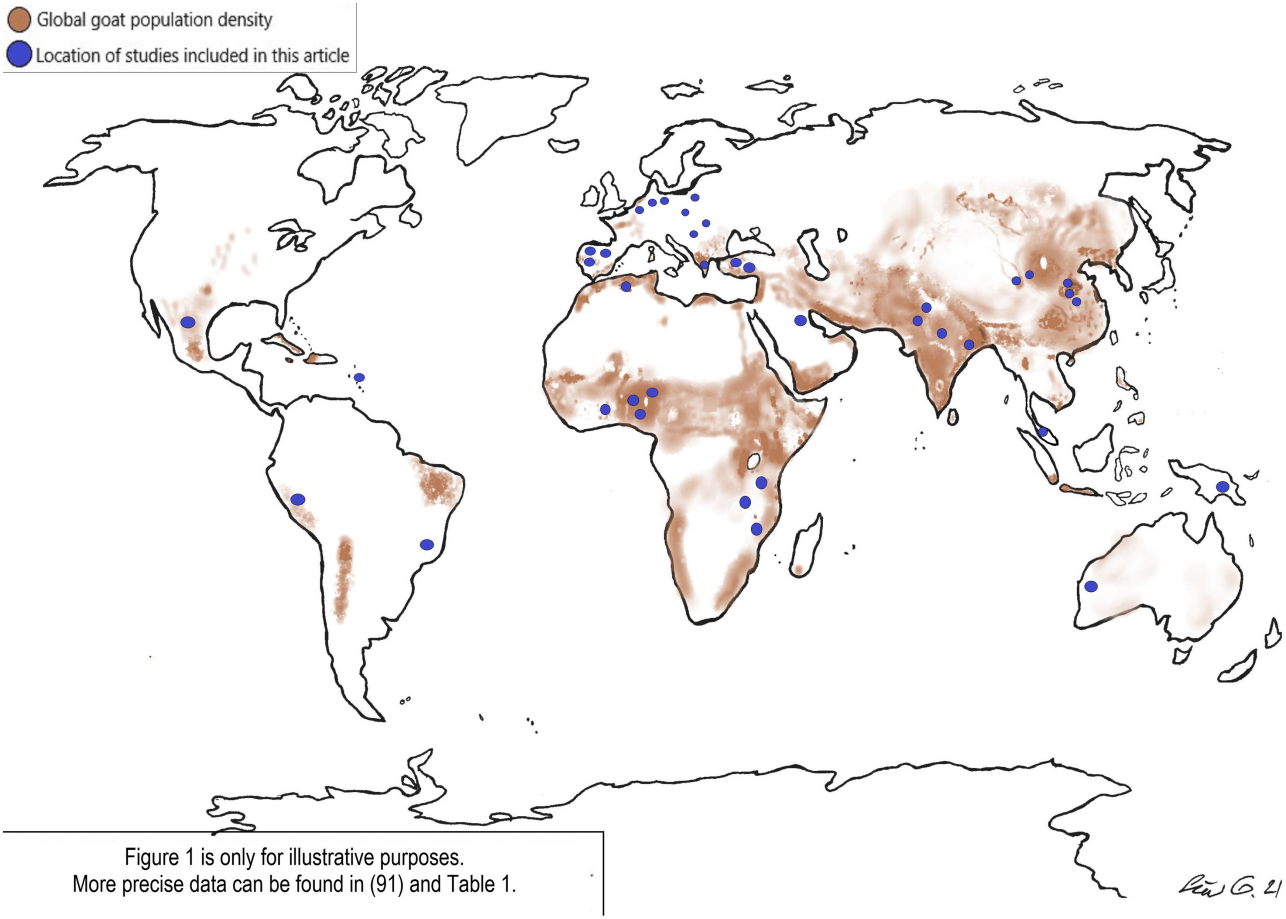
Global goat distribution and locations of studies.

Over two-thirds of goats can be found in subtropical and tropical countries [(6); Figure 1].

In low-income countries of e.g., Asia, Africa, and Latin-America, locally adapted goat breeds are raised for milk and meat, and in dry and drought-prone areas, goat milk is often the only protein source in children's diets (7). In countries where the majority of goats are found, most goat owners belong to the lower socioeconomic strata (8–12), and, in rural areas, goats are largely managed by women and children (13, 14).

The International Livestock Research Institute recognized that goats are more important than cattle to the livelihoods of the rural poor, so investments in goat health, productivity, and sales may greatly assist with poverty alleviation.

### The Cultures of Goat Keeping in Asia and Africa

Where extensive grazing is a main source of livelihood, goats have become an essential aspect of culture, social life, and even, in some places, religion, as goat meat is acceptable according to most scriptures.

Asia has identified the dairy-goat husbandry as especially sustainable in the face of climate change, and investments in several dairy-goat projects have been made during the past decade (7).

In India, for example, domesticated goats account for 20% of the global goat population (15) and goats remain a vital, but under-resourced and denigrated, part of the economy (7). Goats are an integral component in Indian livelihoods, contributing significantly to the income and socio-economic structure of rural farmers, and are often referred to as “the poor man's cow” (16). Goats are a reliable livelihood source in a range of Indian terrains, from deserts to coastal areas and high altitudes. However, unlike other sectors of Indian animal husbandry, the goat-meat industry is relatively disorganized, and abattoirs are usually unsuitable for goat slaughter.

Furthermore, goat husbandry in India takes place under federally unchecked conditions, particularly in rural areas. Regulatory bodies associated with commercial livestock rearing are lacking and most veterinary services inadequate, focusing on treatment rather than preventive measures (17), and gastrointestinal parasitism is prevalent in goats from all areas of India, representing a major health issue (18–20).

Africa holds over 40% of the global goat population, and over 60% are found in sub-Saharan countries. However, in contrast to Asia's relatively positive outlook on goat husbandry, goats are often associated with “backwardness” and “environmental destruction,” by government officials in Africa, making it difficult to gain their investment support (7).

Nevertheless, goats play a major role as a source of food and income, accounting for 30% of Africa's ruminant livestock and producing 17 and 12% of its meat and milk (13). Production systems vary, including smallholder mixed crop-livestock systems, smallholder intensive systems, extensive pastoral and transhumance systems, and large-scale ranching systems (14, 21). Goats in Africa usually graze freely, scavenging feed resources where available, and, during the cropping season, forage for crop residues. The limited management and reliance on children for care and welfare probably exacerbates the low meat and milk production per goat. In urban areas, goats may graze common ground, which is often contaminated and used as a communal latrine, or may be held in stalls and fed at home (14). However, in some parts of East-Africa, there are extensive pastoral and transhumance systems, where goats are reared in large numbers and occupy 50% of the region (22).

## *Cryptosporidium*: an Overview

*Cryptosporidium* is an intestinal protozoan parasite with a worldwide distribution, a fecal-oral lifecycle, and is generally associated with diarrheal disease. It has a direct lifecycle in which the robust infectious oocyst stages are excreted with the feces into the environment and are immediately infectious for the next susceptible host.

### Effects of *Cryptosporidium* on Goat Health

*Cryptosporidium* infection has an impact on growth and production in goats, and has been found to cause anorexia and diarrhea in goat kids, with morbidity and mortality reaching 50 and 100%, respectively (23–27), with accompanying economic consequences, impacting especially marginal farmers. Reduced growth, with and without diarrhea, has also been associated with *Cryptosporidium* infections in goats aged between 9 and 15 months, including in asymptomatic goats, raising further questions regarding long-term effects of apparently asymptomatic infections (28). Some studies have reported asymptomatic shedding of *Cryptosporidium* oocysts in adult goats (29, 30), but the long-term effects of chronic asymptomatic infections remain unclear, and goat health protocols recommended screening for *Cryptosporidium* infections after weaning, even in the absence of diarrhea (28).

### Effects of *Cryptosporidium* on Human Health

Although *Cryptosporidium* has a global distribution, its impact on human health is greatest in developing countries where diarrheal disease exerts a huge health burden. Although global health is steadily improving, diarrheal disease remained the third most common cause of disability-adjusted life-years (DALYs) in the under-10 years age group in 2019 (31).

Given the high prevalence of cryptosporidiosis in people in resource-poor areas, this pathogen was included in the WHO “neglected disease initiative” in 2004 (32).

*Cryptosporidium* infection is particularly associated with pediatric diarrhea (33), but tends to be less important as a diarrheal pathogen in older age groups. A considerable mortality burden from cryptosporidiosis in children younger than 5-years (7.6 deaths per 100,000) has been reported (34), probably reflecting that cryptosporidiosis is acute and the explosive, voluminous diarrhea likely to have a major and immediate impact on infant survival. In addition, *Cryptosporidium* damages cells of the intestine and reduces absorption of nutrients. A meta-analysis suggested that the true burden of cryptosporidiosis was probably underestimated in previous reports, as effects subsequent to the acute phase of infection (decreased growth and enhanced risk of subsequent infections) were not included (35).

## Diagnostic Methods

There are no techniques particularly for diagnosis of *Cryptosporidium* infection in goats, although various procedures are available. Staining techniques are often applied in studies investigating prevalence, and molecular techniques provide information regarding species and subtype. Choice of diagnostic technique depends on available equipment and reagents, analyst experience, and time and cost of analysis. Molecular methods are usually not a routine diagnostic in resource-poor settings, but sensitive and specific diagnostic methods are important everywhere, particularly when positive findings result in appropriate interventions such as improved hygiene and better farm management, both of which can be essential for disease control and prevention in both goats and humans. A recently published study indicated that auramine-phenol staining has high sensitivity and specificity for cryptosporidiosis and can be easily integrated with existing laboratory infrastructures in low-resource settings (36). Targeted sampling and preparation before diagnostics, along with dual application of staining and molecular techniques may provide the best possible results in terms of prevalence and epidemiology investigations.

## Molecular aspects

Molecular tools have changed our understanding of *Cryptosporidium* spp. transmission. Genotyping and subtyping data have clearly demonstrated the presence of anthroponotic, as well as zoonotic, *Cryptosporidium* species in humans in industrialized nations. In contrast, transmission of cryptosporidiosis appears largely anthroponotic in some developing countries; for example, in Africa, despite frequent close contact between humans and animals, transmission appears to be mainly anthroponotic, and human *Cryptosporidium* infection is most often with *C. hominis* or *C. parvum anthroponosum* (37).

Nevertheless, as many *Cryptosporidium* species infect both humans and goats there is clearly the potential for transmission between the two host species. In the overview below, our focus remains on the most common zoonotic types. Details of studies are provided in Table 1, and the location of studies as related to goat distribution is shown in Figure 1.

**Table 1:** Studies investigating Cryptosporidium in goats.

**Occurrence of** ***Cryptosporidium*** **spp. in goats worldwide using different diagnostic techniques**								**Species and subtypes of** ***Cryptosporidium*** **in goats worldwide**			
**Continent and Country**	**Study period**	**No. of goats**	**Goat age**	**Positive numbers of goats according to the diagnostic technique**				**Genes investigated**	***Cryptosporidium* species**	**gp60 genotype**	**References**
				**List of tests used**	**Microscopy**	**Immunological**	**Molecular**				
**AFRICA**											
Algeria	2012–2014	92	4 weeks or younger	PCR			8	SSU) rRNA gp60	*C. ubiquitum* *C. xiaoi*	XIIa	(38)
Ghana	NA	285	0–>24 months	PCR			95	(SSU) rRNA gp60	*C. parvum* *C. baileyi* *C. xiaoi*	IIcA5G3q	(39)
Mozambique	NA	60	Kids	ZN IFA	0						(40)
Nigeria	2013	98	Pre-weaned Post-weaned Adults	ELISA		28					(41)
Nigeria	NA	36	Pre-weaned	ELISA		30					(42)
Tanzania	2010–2011	56	NA	PCR-RFLP			5	SSU) rRNA gp60	C. xiaoi		(43)
Zambia	Na	17	NA	IFA		1					(44)
**ASIA**											
China	2014–2015	629	NA	PCR			104	(SSU) rRNA gp60	*C. parvum* *C. ubiquitum*, *C. xiaoi*	IIdA19G1, IIdA20G1	(45)
China	2007–2013	604	Pre-weaned to adults	PCR			69	(SSU) rRNAgp60	*C. parvum* *C. ubiquitum* *C. xiaoi*	IIaA14G2R1 IIaA15G1R1 IIaA15G2R1 IIaA17G2R1 XIIa	(46)
China	2011–2012	51	NA	IFA PCR		14	8	(SSU) rRNA	*C. parvum*		(47)
China	2006–2007, 2011	1256	Pre-weaned Post-weaned Adult Pregnant Postparturition nannies nannies	Modified acid-fast staining PCR-RFLP	44		44	(SSU) rRNA gp60	*C. ubiquitum* *C. andersoni* *C. xiaoi*	XIIa subtype 2	(48)
China	2006	42 goats + 1 ibex	NA	IFA PCR		15 + 1	2 + 1	(SSU) rRNA	*Cryptosporidium* sp. C. bovis-like genotype *Cryptosporidium* cervine genotype		(49)
India	2016	207	Adults	IFA PCR		1	1	(SSU) rRNA COWP Actin	*C. ubiquitum*		(50)
India	2009–2012	116	>3 months	ZN PCR-RFLP	4		4		*C. parvum*		(51)
India		57	>3 months	ZN PCR-RFLP	2	2	2	(SSU) rRNA Actin	*C. parvum*		(52)
India	NA	20 pooled samples (à 5)	NA	IFA PCR		35 (16–60)	0				(53)
Kuwait	2014–2015	222	Pre-weaned Post-weaned	ZN ELISA PCR-RFLP	22	54	10	SSU) rRNA gp60	*C. parvum* *C. ubiquitum* *C. xiaoi*	IIdA20G1 XIIa	(54)
Malaysia	2015	478	NA	ZN PCR	207		207	(SSU) rRNA	*C. parvum*		(55)
Turkey	2012–2016	NA	10–15 days old, symptomatic	Kinyoun Carbol Fuchsin staining PCR	9		9	(SSU) rRNA gp60	*C. parvum*	IIaA13G2R1 IIaA15G1R1 IIdA22G1 IIdA18G1 mixed subtypes	(56)
Turkey	2016	112	2–4 weeks	IFA PCR		76	73	(SSU) rRNA, gp60	*C. parvum* *C. xiaoi*	lldA18G1 lldA17G1 llaA15G1R1 llaA14G1R1	(57)
**EUROPE**											
Belgium	NA	148	1 day−10 weeks	IFA PCR		14	11	HSP-70 (SSU) rDNA gp60	*C. parvum*	IIdA22G1 IIdA15G2R1	(58)
Czech Republic	2005–2007	26	0.5–4 months	Milacek-Vitovec	2						(59)
France	2012	20 (longitudinal)	Adults	IFA PCR		16	12	(SSU) rRNA	*C. ubiquitum*		(28)
France	2011	35 animals (longitudinal), 254 samples	From birth to weaning	IFAPCR		61	19	(SSU) rRNA	*C. xiaoi* *C. parvum*		(60)
Greece	NA	255	na	IFA		18					(61)
Poland	NA	46	1–7 years old	ZN ELISA	0	0					(62)
Romania	NA	412	One day−6 weeks	ZN	99						(63)
Serbia	NA	88	Up to 90 days old		28						(64)
Spain	2008–2013	118	Up to 5 weeks old	Carbol fuchsin, auramine phenol PCR -RFLP	74		66	SSU rRNA gp60	*C. parvum* *C. ubiquitum* *C.xiaoi*	IIaA13G1R1 IIaA14G2R1 IIaA15G2R1 IIaA16G3R1 IIdA17G1	(65)
Spain	2004–2006	Na/sampled from symptomatic animals	Up to 21 days old	Carbol-fuchsin PCR RFLP	17		17	(SSU) rRNA gp60	*C. parvum*	IIdA17G1a IIdA19G1 IIdA25G1 IIdA26G1	(66)
Spain	2005	184	148 Adults, 36 kids	IFA		A:14 K:11					(67)
Spain	NA	116	Adults, asymptomatic	IFA		9		(SSU) rRNA hsp70	No positives	No positives	(68)
Spain	NA	5	<21 days	Carbol-fuchsin PCR RFLP	2		2	(SSU) rRNA Actin	*C. xiaoi*		(69)
**North America**											
Grenada	2011	202	All age groups	ELISA		45					(70)
**South America**											
Brazil	NA	105	56 >12months 49 <12months	Centrifuge-flotation Safranine Blue	CF:5 SB:2						(71)
Mexico	2014	80	>3 months	ZN	58						(72)
Peru	NA	402	NA	NA				NA	*C. ubiquitum*		(73)
**OCEANIA**											
Australia	NA	125 animals, 500 samples analyzed	9–12 months	PCR			36/500	(SSU) rRNA gp60	*C. ubiquitum* *C. parvum*	XIIa IIaA17G2R1 IIaA17G4R	(74)
Papua New Guinea	2011	228	Adults	PCR			10	(SSU) rRNA gp60	*C. hominis* *C. parvum* *C. xiaoi* *Cryptosporidium* rat-genotype II	IdA15G1 IIaA19G4R1 IIaA15G2R1	(75)

*PCR, Polymerase Chain reaction; RFLP; Restriction Fragment Length Polymorphism; ZN, Ziehl-Nielsen; ELISA, Enzyme-Linked Immunosorbent Assay; IFA, Immunofluorescence Assays; NA, Not available*.

*C. parvum* is perhaps the most studied zoonotic *Cryptosporidium* species. In studies from China in which *C. parvum* infectons from goats were diagnosed and the subtypes determined, the IId-subtype was found (not exclusively) in all investigations. *C. parvum* IId-subtypes seem to have a unique distribution in China, being predominant in *C. parvum* infections in humans, farm animals, and rodents (76–79). The IId-subtype has also been detected in goats in Europe, Asia, and Oceania (Table 1). However, the role of the rodent host, potentially an additional endemic amplifier, remains unknown in these areas.

In Africa, human *C. parvum* infections are dominated by the Iic-subtype, and the role of goats in transmission remains largely unknown. Although a study from Ghana reported finding the Iic-subtype in a goat, non-zoonotic, *C. xiaoi* dominated among goats kept in or around households (80). As far as we know, this is the only study where the Iic-subtype has been found in a goat.

The IIa-subtype seems to be present in *C. parvum* infections in goats in many parts of the world, having been reported from all continents except Africa, and, to date, publications investigating *C. parvum* subtypes in goats in North- and South-America are lacking.

*C. ubiquitum* has been detected in goats in studies from Europe, Asia, Africa, South America and Oceania (Table 1); in studies where subtyping has been conducted, only the subtype-XIIa was found. This subtype seems to predominate in ruminants, and humans are susceptible hosts for subtypes XIIa–XIId (81). *C. ubiquitum* is the most common species found in drinking water in rural USA, and human infections with this species has been detected mostly in developed countries, possibly due to the lower background of anthroponotic infections that predominate in developing countries (82), *C. ubiquitum* has been detected in feces from more animal species, and over a greater geographic range, than most *Cryptosporidium* species – with the exception of *C. parvum* (80). This distribution facilitates establishment of life cycles in extensive farming, where susceptible host animals are likely to be present and the infection barrier is weak. Data on clinical signs is scant, although this species has been identified in many cases of human cryptosporidiosis (81) and it has been isolated from diarrheic goat kids in Spain (65). A French study also found a periparturient rise in *C. ubiquitum* oocyst shedding from asymptomatic nanny goats (29). Although genotype analysis of *C. ubiquitum* has not been extensively performed, this species may represent a greater threat to both humans and animals given its ability to infect its next host, be it humans or their livestock.

### Epidemiological Evidence for Sharing of *Cryptosporidium* Between Goats and Their Keepers

It is well known that younger animals, and people, are at greatest risk of *Cryptosporidium* infection, and are most likely to develop symptomatic disease if infected. Other epidemiological aspects are concerned with routes of exposure, and geographical, meteorological, cultural, and other environmental factors that may affect transmission patterns. Of interest regarding epidemiological pressures for interspecies transmission between goats and people, is looking at where zoonotic transmission from goats to humans has been documented. Although we know that the brunt of the global cryptosporidiosis burdens is borne by populations in Africa, Asia, and Latin America, it is difficult to recognize specific transmission occasions or outbreaks in these countries due to the high prevalence of infections. In other countries, however, outbreaks can be recognized, and some have been associated with direct or indirect contact with goats and their products. For example, an outbreak of cryptosporidiosis in USA was associated with consumption of unpasteurized goat milk (83) and an outbreak of cryptosporidiosis among school children in Norway was associated with contact with lambs and goat kids at a holiday farm, where the same sub-type of *C. parvum* (IIaA19G1R1) was found in both children, lambs, and goat kids (84). It is also noteworthy that in all studies from Table 1 where the species of Cryptosporidium was identified, zoonotic species were detected in all investigations except two.

Of particular relevance regarding goats and *Cryptosporidium* regarding human health, is that in those countries where cryptosporidiosis exerts a particular burden, it is, as previously outlined, children who are most affected; and it is also children who most often have the job of looking after goats in these same regions of the world. The grazing habits of goats, generally browsing on woody shrubs and weeds rather than grazing grass, may indicate that they are less likely to ingest parasites (85). However, in many settings, particularly poor urban or peri-urban areas, where shrubs are scant, they will be forced to search for nutrients closer to the ground. When foraging these scarce food resources on the ground, goats may be more likely to ingest *Cryptosporidium* spp. oocysts contaminating the environment, possibly shed by the human kid tending the goats, or from the goat kid foraging beside it. Similarly, children tending a flock of goat kids are likely to be exposed to parasite transmission stages in goat feces. In the cooler climates of temperate regions bovines, particularly calves, are often considered a source of zoonotic transmission of *Cryptosporidium*; in other global regions, it seems possible that goats may be an even more likely source.

## Prevention and Control

Persistent diarrhea seriously affects nutritional status, growth, and intellectual function. Meeting these challenges is profoundly important, particularly in developing countries. *Cryptosporidium* oocysts have high infectivity, robustness, and resistance to disinfectants, which underscores the need for improved treatment options. No safe and effective treatment for cryptosporidiosis has been identified to date, although efforts to direct resources toward this objective continue to be made (86). Although *C. hominis* apparently still predominates in many settings, zoonotic transmission should not be neglected. In line with the One Health initiative, general rules of hygiene barriers between and among humans and animals in any setting should be implemented and thus reduce infection risks, not only of *Cryptosporidium*, but other zoonotic pathogens as well. As children and women are often responsible for tending backyard livestock, and also usually prepare food and/or fetch water, focusing on this group in hygiene training and information dissemination could improve the wellbeing of both them and their goats beyond their backyard. Studies that focus particularly on the likelihood of transmission of *Cryptosporidium* between goats and their keepers may provide more specific information on where interventions should be targeted, without losing the value from goat-keeping as an important resource for lifting families and communities out of poverty.

## Goats Are Saving the World

Organizations like Heifer International have helped small-scale farmers to obtain and benefit from goats in widely ranging situations, including in the dry forest areas of Peru, landless women in India, tropical forest areas of West-Africa, farmers in peri-urban areas St. Petersburg, the densely populated highlands of East-Africa, as well as the Sichuan province in China. Most of these goats are kept in small flocks of 3–10 animals, and are mainly cared for by children and women. Women have a significant role in goat-keeping in rural areas, enabling them to contribute substantially to the household economy (87).

In a resource-poor region of northern India, goat prices almost doubled when low-cost shelters, feeders, and water sheds were provided, in addition to improved breeding practices and prophylactic measures (7). Other development projects with goat interventions have given a positive return rate for both small- and large-scale goat-keepers in both Africa and South America, which, in turn, increased their income substantially (88). A zero-grazing management practice has often been introduced, which involves keeping goats in pens with limited outdoor space for exercise and all feed being brought to them. Manure is collected and either composted or applied to crops (89). This system has proven very successful in disease control, breeding management, and goat-rearing integration, including better protection of natural resources (90). However, the application of manure to crops might impose potential health risks and appropriate measures to protect both farmers and ensure safe produce should be taken into consideration.

The socio-economic status of farmers plays a major role in flock size and adoption of scientific management practices for goat rearing, which thereby raises income and socio-economic level of the owner, and particularly benefits socio-economically deprived women.

## Conclusion

Cryptosporidiosis is an important diarrheal illness; in people in developing countries it exerts a substantial burden on child health, growth, and development (35) and in ruminant livestock, including goats, it affects growth and production (28). With goats an important livestock species for under-resourced communities, it is important to ensure that this potential reservoir of zoonotic *Cryptosporidium* is addressed and managed, and research needs to be conducted in the relevant regions.

The One Health initiative, focusing on reducing disease interface between humans and animals in areas where infection risk is greatest, could be harnessed to reduce health burdens and economic challenges where most needed. This depends largely on local endemic status and appropriate interventions. Studies on prevalence and species/genotypes of *Cryptosporidium* infecting people in developing countries are extensive, but there are considerably fewer of such investigations among domestic livestock. More information provided through further epidemiological studies on the species of *Cryptosporidium* infecting livestock and humans in these regions will fill data gaps and may assist in pinpointing relevant approaches to minimizing transmission. Goat-keeping is often a trade for the poorest in society, and awareness of proper hygienic routines and appropriate animal management strategies could benefit both human and animal health, as well as improving the economy and welfare of the goat-keepers and their herds.

## Author Contributions

KU conceived the study and wrote the main bulk of the manuscript. SC and TK contributed significantly with local knowledge regarding both epidemiology and animal husbandry in the manuscript. LJR structured and contributed to all parts of the manuscript. All authors contributed to the article and approved the submitted version.

## Conflict of Interest

The authors declare that the research was conducted in the absence of any commercial or financial relationships that could be construed as a potential conflict of interest.
